# COVID-19 lockdown in Italy: the role of social identification and social and political trust on well-being and distress

**DOI:** 10.1007/s12144-020-01141-0

**Published:** 2020-10-26

**Authors:** Daniele Paolini, Fridanna Maricchiolo, Maria Giuseppina Pacilli, Stefano Pagliaro

**Affiliations:** 1 University of Roma Tre, Rome, Italy; 2 University of Perugia, 06123 Perugia, PG Italy; 3 University of Chieti-Pescara, Chieti, Italy

**Keywords:** COVID-19, Social identification, Trust, Well-being, Distress

## Abstract

The COVID-19 pandemic has rapidly become a global health crisis, leading people to change their interpersonal behaviours to contain the spread of the virus. Italy has rapidly become the country hit second hardest in the world by the COVID-19 pandemic and the first one in Western countries. To reduce the spread of the COVID-19, people are required to change their interpersonal behaviours, reducing their social interactions in close contacts. The lockdown impact on the economy as well as on social and psychological processes is relevant, we conducted an exploratory study to examine which social factors are associated with the psychological reactions of Italians during the COVID-19 lockdown. Participants (*n* = 690) self-reported their social identification on three levels (i.e., Italians, Europeans and humankind), their trust toward social and political actors, and their level of welbeing, interdependent-happiness, and distress. Results showed that the relation between trust and the level of wellbeing and distress was mediated by identification with Italians and humankind, only the identification with humankind mediated the relationship between trust and the level of interdependent-happiness. The identification with Europeans did not emerge as a mediator in such relationships. The implications for dealing with COVID-19 lockdown in Italy are discussed.

Since December 2019, a new acute respiratory syndrome in humans has emerged in Wuhan, China (i.e., Covid-19), causing a rapid spread of over 118,000 cases and over 4000 deaths in 114 countries in just three months (Zhou et al. 2020). This emergency led the World Health Organization (WHO) to declare a global pandemic, leading to a massive global public health campaign to reduce the spread of the COVID-19 through compliance with specific indications and restrictions (i.e., increasing handwashing, reducing face touching, wearing masks in public, and physical distancing; Van Bavel et al. 2020).

At the beginning of the virus spread in Europe, Italy has rapidly become the country hit second hardest in the world by the COVID-19 pandemic and the first one in Western countries. As a consequence, the Italian government has imposed a state of emergency lockdown, which previously started in northern Italy and then, on the 10th March 2020, has expanded to the whole country. It lasted until 4th May 2020 to contain a contagion that, at the time of writing, has infected over 240.436 people and killed 34.744 Italians, while the infected around the world exceed 10mln persons and over 500.000 dead (www.worldometers.info).

The lockdown impact on the economy as well as on social and psychological processes is relevant, and an increasing number of socio-psychological studies have been conducted around the world on this topic while we are writing. The Covid-19 pandemic led people to experience a severe level of anxiety, fear, and worry (Cao et al. 2020; Kachanoff et al. 2020), sleep difficulties, depression (Cellini et al. 2020), panic and stress disorder, psychological trauma – PTSD (Qiu et al. 2020) and difficulty in emotions control (see Van Bavel et al. 2020) as well as concerns for the own and close others health and the future (see World Health Organization 2020). Since research on Covid-19 emergency has suggested that the social support helps people to face with and to reduce the psychological pressure (Cao et al. 2020; Chen et al. 2020), a deeper understanding of which social factors predict the psychological reactions of people during the COVID-19 lockdown is needed.

To reduce the spread of the COVID-19, people are required to change their interpersonal behaviours, reducing their social interactions in close contacts. While on the one hand, this emergency condition preserves the physical health of people, on the other hand, it also threatens the satisfaction of their basic social needs. Indeed, people are social beings who are strongly motivated to belong to social groups and to interact with others in order to gain and preserve security, well-being, and high self-esteem (Baumeister and Leary 1995). Moreover, individuals identify with groups for many other reasons, for example, the fulfillment of self-enhancement motivation (Tajfel and Turner 1979), the need for optimal distinctiveness (Brewer 1991), or the need for reducing subjective uncertainty (for a review, see Hogg 2000). Thus, individuals may define themselves in terms of their individual characteristics, emphasizing what makes them unique, and in terms of the belongingness, emphasizing what makes them similar and interchangeable with others (i.e., Social Identity Theory; Tajfel and Turner 1979). This dual perspective suggests that the social identity not only plays a crucial role at the individual level of well-being (e.g., stress reactions, or satisfaction with life, Diener et al. 1985) but also at the interdependent level, that is, happiness based on social relationships, focusing on the relational nature of human beings (Hitokoto and Uchida 2015). Individuals’ well-being is related to the relationships they establish with closer individuals as well as social structures, physical environments, and with their own culture (Maricchiolo et al. 2020a). Well-being is often described as happiness, which represents the highest value and the supreme goal of society (Lu and Gilmour 2004). Well-being is emergent, the outcome of accommodation and interaction that happens in and over time through the dynamic interplay of personal, societal, and environmental structures and processes (White 2017; Maricchiolo et al. 2020b).

In sum, social relationships and the social identities that people derive from social groups represent the “social core” that presents important consequences for their health and well-being (Haslam and Loughnan 2014; Haslam et al. 2009; Jetten et al. 2014; Jetten et al. 2017).

## Study Hypotheses

Social psychologists can provide crucial insights to understand the impact of the COVID-19 pandemic on the population (see Van Bavel et al. 2020). Literature investigating the impact of previous pandemics (e.g., Ebola virus in Liberia; Blair et al. 2017) suggested that social, cultural, and political factors may be decisive in fighting the spreading the virus, beyond the epidemiologic factors. Specifically, research highlighted that the trust in governments/institutions plays a crucial role in both citizens’ compliance with public health policies, restrictions, and guidelines, and in their health (see also, Chowell and Nishiura 2014; Hewlett and Amola 2003). Based on the Social Identity Theory (Tajfel and Turner 1979), one way to investigate the initial impact of the COVID-19 lockdown is to understand whether the relation between people’s trust toward social and political actors and people’s well-being (and distress) is mediated by social identification.

“*We are not here to close spreads, there are other tools and other actors to deal with these issues…*” were the words pronounced by Christine Lagarde – President of the European Central Bank – during her public speech on 13th March 2020 when only Italy was striving with a severe wave of COVID-19 infections. These words provoked not only a negative impact on the Italian economy, but also on the Italians’ level of belonging toward Europe, probably leading social and political actors to trigger a superordinate social categorization among Italians, such as humankind, to face with the negative impact of COVID-19.

Based on these premises, in an explorative vein, we tested the mediational role of the increasing level of abstraction of social identification, such as Italian, European, and humankind, on the relation between Italians’ trust toward social and political actors and both their level of well-being (individual and interdependent) and distress, as a form of “unwell-being”. Thus, we explored whether and how identification at three different levels of abstraction mediated the relationship between trust and well-being and between trust and distress.

## Method

### Participants

We recruited 713 Italian participants by spreading an online survey through Social Networking Sites (i.e., SNS). Therefore it is not a statistically representative selected sample. Participants took part in the survey on a voluntary basis. Twenty-three participants were excluded from the analyses because they did not provide their consent to participate. Therefore, the retained sample consisted of 690 Italian participants (231 males, 455 females, 4 missed to declare gender; *mean age* = 38; *SD* = 14.85).

The sample was recruited from the Northern (*n* = 103; 15%), Central (*n* = 288; 42%), and Southern (*n* = 299; 43%) regions of Italy. They declared to live in the centre of a big city (*n* = 119; 17%), in a suburban area of a big city (*n* = 136; 20%), in a small or medium inland city (*n* = 215; 31%), in a small or medium coastal city (*n* = 154; 22%) and in a rural area (*n* = 65; 10%).

Furthermore, participants declared to face with the COVID-19 lockdown period alone (*n* = 66; 10%), with one other person (*n* = 147; 21%) and together with others: From 2 to 5 persons (*n* = 444; 64%), from 6 to 10 persons (*n* = 28; 4%) and more than 10 persons (*n* = 5; 1%).

### Procedure

The questionnaire was implemented by using the Google Forms platform. The survey was alive during a brief period of the COVID-19 lockdown in Italy (i.e., from 1st April 2020 to 4th April 2020). Participants were recruited by snowball sampling posting the survey link on social networks.

The questionnaire took approximately 15 min to fill in. According to the ethical standards Declaration of Helsinki (World Medical Association, 2001), participants were informed about all relevant aspects of the study (e.g., methods, institutional affiliations of the researchers) before they started to fill out the questionnaire. Importantly, they were apprised of their right to anonymity, to refuse to participate in the study, or to withdraw their consent to participate at any time during the study without fear of reprisal. Participants then confirmed that they had understood the instructions correctly, agreed to participate, and began filling out the questionnaire. The research protocol was approved by the local Ethics Committee of the first author’s institution.

### Materials

#### Trust toward Social and Political Actors

Participants were asked to report their level of trust toward 9 specific social and political figures and institutions through a built ad-hoc scale (i.e., “National Health System”; “Prime Minister”; “police”; “politicians”; “scientists”; “Civil Defence” and “journalists”) on a Likert-type scale from 1 (*No trust at all*) to 9 (*Maximum trust*). We averaged responses to all these measures to create an overall Trust index (alpha = 0.77). Higher ratings revealed more trust toward social and political actors.

#### Social Identification

Participants answered 3 different social identification built ad-hoc scales, on a Likert-type scale from 1 (*It doesn’t describe me at all*) to 9 (*It describes me exactly*). The first one indicated the participants’ level of social identification with the Italian ingroup. The second one is referred toward the European ingroup. In the last one, participants indicated their level of identification with humankind. Each scale was composed by the same 4 items (i.e., “*Being Italian/Being European/Belong to humankind reflects what I am*”; “*Being Italian/Being European/Belong to humankind is an important part of the image of myself*”; “*Sometimes being Italian/being European/belong to humankind bothers me*” and “*Being Italian/Being European/Belong to humankind makes me feel good*”; alpha = 0.76, 0.78 and 0.74, respectively). We averaged responses for each scale – after reverse-coding negative items – to create 3 different social identification indexes. Higher ratings indicated stronger identification respectively with the Italian ingroup, the European ingroup, and the humankind.

#### Individual Well-Being

Participants’ level of well-being was assessed by using the *Satisfaction with Life Scale* (SWLS; Diener et al. 1985). Participants filled out 5 items (e.g., “The conditions of my life are excellent”; “I am satisfied with my life; alpha = 0.87) ranging on a Likert-type scale from 1 (*It doesn’t describe me at all*) to 9 (*It describes me exactly*). We averaged responses to create an overall individual well-being index, in which higher ratings indicated higher individual well-being.

#### Interdependent Happiness

We then asked participants to complete the *Interdependent Happiness Scale* (IHS; Hitokoto and Uchida 2015), which measures happiness based on harmony with others, in particular with own groups. They answered 9 items (e.g., “I believe that I and those around me are happy”; “I feel that I am being positively evaluated by others around me”; alpha = 0.87) ranging on a Likert-type scale from 1 (*It doesn’t describe me at all*) to 9 (*It describes me exactly*). We averaged responses to these items to create an overall interdependent happiness index, in which higher ratings indicated higher interdependent happiness.

#### Distress

To evaluate the participants’ level of general distress, we used the *Depression Anxiety Stress Scales-21* (DASS; Bottesi et al. 2015). Originally, it contains 21 items that are grouped into 3 subscales assessing people’s level of depression (7 items; “I felt discouraged and depressed”); Anxiety (7 items; “I realized that my mouth was dry”) and stress (7 items; “I have tended to overreact to the situations”). For technical issues, responses to one item were not recorded. Participants thus filled out the 20 items of DASS by thinking of how they felt during the COVID-19 lockdown on a Likert-type scale from 1 (It never happened to me) to 9 (It almost always happened to me). To create a single index for general distress (alpha = 0.95), we averaged responses for the three subscales scores. Higher ratings indicated a higher level of depression, anxiety, and stress, overall.

#### Socio/Demographic Questions

Participants finally provided socio/demographic information that included their age, gender, the place of belonging in Italy, and the size of their living area in Italy. They also reported how many people they faced the COVID-19 lockdown with.

## Results

Table 1 shows the means and standard deviations among all variables and the correlations between all measures investigated in the study. In order to test our exploratory hypotheses, we conducted a series of mediation analyses by using the SPSS macro developed by Hayes and Preacher (2014). According to the literature and the rationale described above, we tested 3 different mediation models (PROCESS model number 4) in which trust toward social and political actors was modelled as independent variable, the three social identifications were modelled as parallel mediators, and the participants’ level of individual well-being; interdependent happiness and distress as dependent variables. In the first model, we investigated the mediation role of the three different social identifications (i.e., Italian ingroup, European Ingroup, and humankind) in the relation between participants’ trust toward social and political actors and their individual well-being. In the second model, we tested the mediation role of the three different social identifications in the relation between participants’ trust toward social and political actors and their interdependent happiness. In the last model, we verified whether the relation between trust toward social and political actors and the participants’ level of distress was mediated by the three different social identifications.

**Table 1:** Means (standard deviation) and zero-order correlations among variables (*n* = 690)

	Means (SD)	1.	2.	3.	4.	5.	6.	7.
1. Identification with Italians	5.81 (1.78)	1.00						
2. Identification with Europeans	5.10 (1.91)	.204**	1.00					
3. Identification with humankind	7.51 (1.53)	.343**	.244**	1.00				
4. Trust toward social and political actors	5.68 (1.22)	.400**	.281**	.299**	1.00			
5. Individual wellbeing	5.83 (1.58)	.244**	.176**	.292**	.314**	1.00		
6. Interdipendent Happiness	5.76 (1.37)	.201**	.139**	.259**	.277**	.731**	1.00	
7. Distress	3.13 (1.57)	−.030	−.091*	−.089*	−.044	−.242**	−.304**	1.00

Note: **p < 0.01; *p < 0.05

### Individual Well-Being

**Fig. 1:**
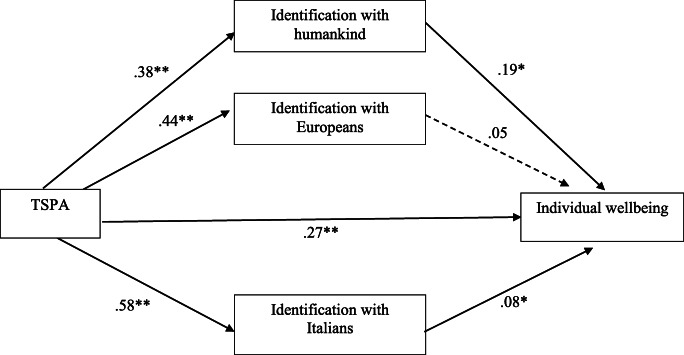
Identification with the Italians and humankind jointly mediate the effect of trust towards social and political actors (TSPA) on the individual wellbeing [Note: **p < .001; *p < .05]

The model in which the effect of the participants’ trust toward social and political actors on individual wellbeing (i.e., SWLS) was mediated by the three different social identification was significant (*R*^*2*^ = 0.15; *F* (4, 685) = 30.43, *p* < 0.001; see Fig. 1). The bootstrap analysis with 5000 resampling showed that indirect effects via the identification with the Italian group (*b* = 0.04; 95% CI: LLCI = 0.0014; ULCI = 0.0890) and via the identification with the humankind (*b* = 0.07; 95% CI: LLCI = 0.0401; ULCI = 0.1144) were significant, while the indirect effect via the identification with the European group (*b* = 0.02; 95% CI: LLCI = −0.0058; ULCI = 0.0480) was not significant. The direct effect considering the mediators was still significant (*b* = 0.27; 95% CI: LLCI = 0.1699; ULCI = 0.3729).

The test of this first model revealed that the social identification with the Italian group and with the humankind mediated the relation between participants’ level of trust toward social and political actors and their individual well-being. Furthermore, such relationship was not mediated by the participants’ social identification with European group.

### Interdependent Happiness

**Fig. 2:**
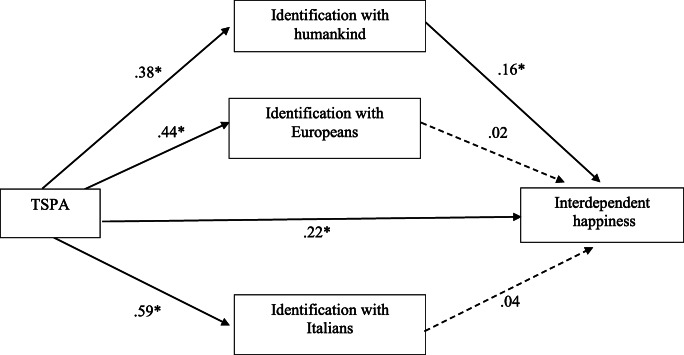
Identification with humankind alone mediate the effect of trust towards social and political actors (TSPA) on the interdependent happiness [Note: *p < .001]

The overall equation was significant (*R*^*2*^ = 0.11; *F* (4, 685) = 22.17, *p* < 0.001). As shown in the Fig. 2, a bootstrapping procedure with 5000 resamples revealed that the indirect effect of participants’ level of trust toward the social and political actors on their interdependent happiness (i.e., IHS) was only mediated by the identification with humankind (*b* = 0.06; 95% CI: LL = 0.308; UL = 0.935). The indirect effect via the identification with Italians (*b* = 0.03; 95%, CI: LL = −0.0133; UL = 0.0647) and Europeans (*b* = 0.01; 25% CI: LL = −0.0139; UL = 0.0336) were not significant. The direct effect considering the mediators was still significant (*b* = 0.22; 95% CI: LLCI = 0.1284; ULCI = 0.3084).

As regards the second model, the analysis showed that only the social identification with humankind – and not with Italian and European group – mediated the relation between participants’ level of trust toward the social and political actors and their level of interdependent happiness.

### Distress

**Fig. 3:**
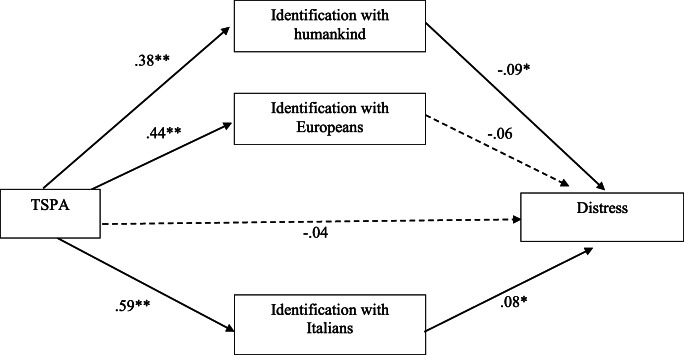
Identification with the Italians and Humankind jointly mediate the effect of trust towards social and political actors (TSPA) on Distress [Note: **p < .001; *p < .05]

The overall equation was significant (*R*^*2*^ = 0.02; *F* (4, 685) = 3.38, *p* = 0.01; see Fig. 3). The bootstrap analysis with 5000 resampling showed that indirect effects of participants’ level of trust toward the social and political actors on their level of distress via the identification with the Italian group (*b* = 0.04; 95% CI: LLCI = 0.0045; ULCI = 0.0909) and via the identification with the humankind (*b* = −0.03; 95% CI: LLCI = −0.0700; ULCI = −0.0060) were significant, The indirect effect via participants’ identification with the European group (*b* = −0.03; 95% CI: LLCI = −0.0610; ULCI = 0.0007) was not significant, as well as, the direct effect of trust considering the mediators (*b* = −0.04; 95% CI: LLCI = −0.1478; ULCI = 0.0687).

The analysis of the third model indicated that the social identification with the Italian group and with the humankind mediated the relation between participants’ level of trust toward the social and political actors and their level of distress, while the social identification with the Europeans group did not mediate such relationship. Identification with Italians positively related with distress.

## Discussion

Italy was one of the first countries to face the crisis worldwide of the COVID-19 disease, the first one in Europe to carry out the lockdown as a virus containment strategy. Lockdown has been having a profound impact both on individuals’ quality of life and well-being as well as emotional and affective stability, due to the uncertainty of the situation and the end of the pandemic (Cao et al. 2020; Cellini et al. 2020; Kachanoff et al., in press; World Health Organization 2020; Qiu et al. 2020; Van Bavel et al. 2020).

The results of this cross-sectional study, conducted in full epidemic crisis in Italy, showed the critical role of social identification for the relation between trust toward institutional structures and political decisions, during such crisis, and well-being or “unwell-being”.

Regarding well-being, we found trust positively related with well-being and interdependent happiness of people by social identification with a specific group. In particular, Italian social identity and identification with humankind mediated such a positive relation with individual satisfaction with life, whereas only social identification with humankind mediating the positive relation between trust with interdependent happiness. This is probably due to the relational and reciprocal nature of interdependent happiness, which relates more to identification to a more abstract group, or better a superordinate category which was attacked by the COVID-19. We must remember that Italy was the first country in Europe hit by the epidemy, the first to implement the lockdown, and with the highest incidence rate of the disease in Europe, at the time of data collection. This meant that identification with the Italian group has positively related to the trust and has positively impacted on personal well-being, but not with interpersonal well-being, but also on distress, probably because, as Italians, people felt more unlucky compared to other Europeans and this may have adversely affected their emotional and affective state of the moment. While belonging to a broader category such as humankind, as a sense of union and solidarity with all individuals vulnerable to the virus, had a positive impact with well-being, happiness and negative impact with distress. The role of identification with Europeans is a worth-noting aspect of our findings. Despite the high positive relationship between trust and the identification to Europeans, this has no relationship either with well-being nor happiness, nor with distress; therefore it does not mediate the relationship between trust and outcomes. This could be due to Europe initial reactions to the COVID-19 problem in Italy. Both the other European Union (i.e., EU) member countries and the EU institutions themselves, through the media, conveyed a reaction of distance or indifference with what was happening in Italy, in particular the words “*We are not here to close spreads, there are other tools and other actors to deal with these issues…*” pronounced by Christine Lagarde – President of the European Central Bank (i.e, ECB) – during her public speech on 13th March 2020 when only Italy was striving with a severe wave of COVID-19 infections. Not only these words shook the Italian government bond market who lost 17% that specific day, but also provoked a general outrage of Italian public against Lagarde and ECB opinions’ level of belonging toward Europe, leading social and political actors to trigger a superordinate social categorization among Italians, such as humankind, to face with the negative impact of COVID-19. But it could also explain positive relation between Italian social identity and distress.

A possible interpretation could be found in the group that the Italians participants each time identified as outgroup in their different social categorizations. In periods of social crisis, trust in politics and social institutions, which should be able to overcome the crisis, therefore the enemy (the outgroup) who provoked it, strengthens identification with the social group in crisis (see, Blair et al. 2017). In this specific case, while it is not certain that categorization with the Italians Group makes perceive Europe as an outgroup, it is very probably that identification with humankind makes perceive the coronavirus as the outgroup and this would explain the high averages of the score (Identification with humankind *M* = 7.52) in a historical moment represented in the social imagination as a War whose enemy was COVID-19. Feeling part of the same destiny with a larger and inclusive group that fights (or defends) against the same enemy would therefore positively mediate the positive relationship between trust in institutions and politics and the positive psychological reaction to such a crisis due to a pandemic, which threatens not only physical health but also undermines social and individual stability of the community.

This study has some relevant limitations, starting from the cross-sectional nature of the data. Nevertheless, even though we should be cautious about the causal interpretation of our findings, previous literature sustains the relations we tested. This is true with regard to both the relations between uncertainty/crises and ingroup identification and the mediation role of social identification on individual’s stress and happiness. Moreover, the snowball sampling procedure did not allow us to consider our sample as a representative one, even though it covered almost all the Italian geographical areas and thus may well represent the general public. Future studies could investigate other social factors that could predict the psychological reactions of Italians after the COVID-19 lockdown phase. In this vein, one could investigate the role of group entitativity and group-based resiliency (see, Pagliaro et al. 2013).

In conclusion, it is well known that the lockdown has been having a profound impact both on individuals’ quality of life and well-being. However, our results suggest that, during uncertainty and in a threat condition such as a pandemic, focusing on a larger and inclusive group identification can play a strategic role in fostering a more effective relationship between the trust toward social and political actors and individual and interpersonal well-being. Trigger on a resilient mechanism could be a crucial way to face with both the emergency phase and the challenge of the (re)opening phase.
